# Implementation of ensemble machine learning algorithms on exome datasets for predicting early diagnosis of cancers

**DOI:** 10.1186/s12859-022-05050-w

**Published:** 2022-11-18

**Authors:** Abdu Rehaman Pasha Syed, Rahul Anbalagan, Anagha S. Setlur, Chandrashekar Karunakaran, Jyoti Shetty, Jitendra Kumar, Vidya Niranjan

**Affiliations:** 1 Department of Information Science and Engineering, RV College of Engineering, Bangalore, 560059 India; 2 Department of Computer Science and Engineering, RV College of Engineering, Bangalore, 560059 India; 3 Department of Biotechnology, RV College of Engineering, Bangalore, 560059 India; 4 Bangalore Bio-Innovation Centre (BBC), Helix Biotech Park, Electronic City, Phase-I, Bangalore, 560100 India

**Keywords:** Cancer, Ensemble machine learning, Exploratory data analysis, SMOTE, GAN, TVAE

## Abstract

Classification of different cancer types is an essential step in designing a decision support model for early cancer predictions. Using various machine learning (ML) techniques with ensemble learning is one such method used for classifications. In the present study, various ML algorithms were explored on twenty exome datasets, belonging to 5 cancer types. Initially, a data clean-up was carried out on 4181 variants of cancer with 88 features, and a derivative dataset was obtained using natural language processing and probabilistic distribution. An exploratory dataset analysis using principal component analysis was then performed in 1 and 2D axes to reduce the high-dimensionality of the data. To significantly reduce the imbalance in the derivative dataset, oversampling was carried out using SMOTE. Further, classification algorithms such as K-nearest neighbour and support vector machine were used initially on the oversampled dataset. A 4-layer artificial neural network model with 1D batch normalization was also designed to improve the model accuracy. Ensemble ML techniques such as bagging along with using KNN, SVM and MLPs as base classifiers to improve the weighted average performance metrics of the model. However, due to small sample size, model improvement was challenging. Therefore, a novel method to augment the sample size using generative adversarial network (GAN) and triplet based variational auto encoder (TVAE) was employed that reconstructed the features and labels generating the data. The results showed that from initial scrutiny, KNN showed a weighted average of 0.74 and SVM 0.76. Oversampling ensured that the accuracy of the derivative dataset improved significantly and the ensemble classifier augmented the accuracy to 82.91%, when the data was divided into 70:15:15 ratio (training, test and holdout datasets). The overall evaluation metric value when GAN and TVAE increased the sample size was found to be 0.92 with an overall comparison model of 0.66. Therefore, the present study designed an effective model for classifying cancers which when implemented to real world samples, will play a major role in early cancer diagnosis.

**Supplementary Information:**

The online version contains supplementary material available at 10.1186/s12859-022-05050-w.

## Introduction

### Background of study

The coding part of the genome is referred to as an exome. Any genetic abnormalities in the exomes are known to trigger several types of cancers. With the present prevailing cancer scenario in the world on a constant uprise, extensive research is being carried out to arrive at possible solutions for early diagnosis [1–3]. With possible early diagnosis of the disease and application of suitable treatment strategies still hazy in research, there is an urgent need for the design and development of alternative ways that provide faster and precise predictions via comprehending the huge amount of existing cancer data. One important approach is to develop a decision support system (DSS), which predicts patient specific cancer probabilities, and overcomes challenges that arise with wrong treatment decisions and prognosis, massive data interpretation and comprehending patient-specific causes [4]. As an emerging and ever-evolving technology, DSS systems are highly adept at improving the decision-making process, thereby providing support to clinicians and diagnosticians [5]. Currently, there are several approaches to classify the cancer types, based on the exome datasets that are essential for designing a decision support system (DSS) for early diagnosis of cancers [6–9]. With advent of technology, using artificial intelligence and machine learning on high-throughput data to design an improved DSS model is the premise of the present study.

### Related works

Classification algorithms such as support vector machines (SVM), K-nearest neighbors (KNN), Naïve Bayes, decision trees and random forest are primarily being used for cancer classification using machine learning [10, 11]. Studies have previously classified cervical cancer datasets [12] using KNN and SVM, breast cancer using decision tree algorithm [13] and brain tumor classification and detection using decision trees and KNN [14]. Likewise, the use of conventional ML algorithms such as random forests, decision trees, KNN, artificial neural networks, and SVMs were shown to produce positive results in the classification of lung, prostate, breast, colorectal and gastric cancers, using clinical and genomic data [15]. Despite this, more advanced techniques are being sought after for attaining an overall precision and reliability of the decision support model. Ensemble methods is one such advanced technique, wherein, more than one single method will be integrated to obtain a solution for the same problem [16, 17]. The main advantage of using this approach is that it overcomes the drawbacks of using single algorithms and in turn, consolidates its strengths [18]. Due to this reason, researchers have begun to utilize this technique, particularly to classify various cancer types [18, 19]. Recent studies have employed this technique in an attempt to assist the diagnosis of cervical cancer [20], and breast cancer [21, 22]. Keymasi et al. [23], studied three ensembles of SVM, ANN and KNN to predict and classify he cervical cancer related images and Zhang et al. [24], proposed to classify the benign and malignant breast tumors using an ensemble machine learning model by combining SVM, KNN and decision tree algorithms. As is observed in these studies, ensemble learning has been used to classify depending on imaging data and for specific cancer types alone. However, not many studies exist that focus on classifying several different cancer types, in a single ensemble-based model, as is the case in the present study.

### Research gap

Our research work also uses previously obtained [25, 26] novel genomic data in the form of mutation information for each of the twenty exome datasets to classify these cancer types, making the study unique when compared to previously published works, where the focus of the model has been to use imaging and already available clinical data. Bearing this in mind, our research aimed to address the problem of noise and class-imbalance in twenty cancer exome datasets, derived from our previous work Padmavathi et al. [25, 26] and as an improvement of a DSS model previously designed, by employing various machine learning algorithms with a greater focus on the implementation of ensemble machine learning on the derivative datasets, alongside use of techniques such as oversampling to attain a balanced dataset.

Additionally, a decision support system is generally categorized into three types- model driven, knowledge driven, and data driven [27, 28]. The present study focused on developing a model-driven decision support system from previously gathered data, with an emphasis on reducing the high-dimensionality of the dataset, obtaining a derivative dataset and to improve upon the model training time along with reduced correlation among the features of the dataset. This research work aimed to fulfill the lacunae of creating an all-in-one model from appropriate derivate datasets, from many different cancer types, which has not been previously carried out. Additionally, efforts were also focused on obtaining an appropriate derivative dataset from the raw data, that could eventually help in reducing the calculation inefficiencies and provide better predictions on the weighted features alone.

### Contribution of present study

The major contribution of our study is towards the development of a highly accurate and improved decision support model, which when used in healthcare, will provide immense benefits to the diagnosis and control of cancers. Additionally, our model encompasses classifications and predictions for five cancer types, making it a novel study with huge potential for early diagnosis of five different cancer types. The reduction of dimensions in the datasets were covered in our study to derive an appropriate derivative dataset which is of utmost importance since they directly contribute to providing better and more accurate predictions on the features of importance. The present study also provides massive insights into the workings of our proposed model, which resulted in a much better overall accuracy when compared to similar such previous work, satisfying the rudimentary aim of our research work, to offer support to the management of healthcare.

## Materials and methods

**Fig. 1:**
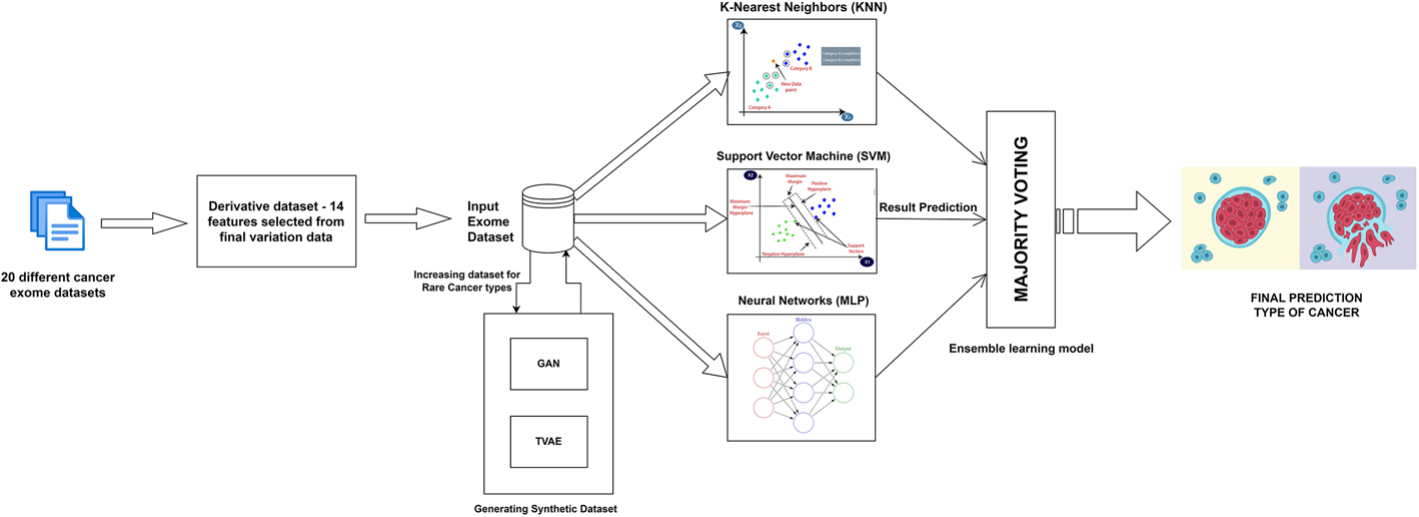
Block diagram summarizing the workflow from cleaning and obtaining the derivative exome datasets, using classification analysis by including three classifiers such as KNN, SVM and a multi-layer perceptron network. This was followed by using majority voting-based ensemble classifiers to obtain the expected results

A block diagram summarizing the proposed work, from cleaning and obtaining the derivative exome dataset, using classification analysis by including three classifiers, namely, K-NN, SVM, and a multilayer perceptron network. This was then followed by using majority voting-based ensemble classifier, to finally obtain the proposed results (Fig. 1).

### Dataset analysis

A preliminary analysis of the exome datasets was carried out. These datasets were obtained after a careful analysis of twenty cancer exome datasets, belonging to five cancer types, obtained from our previous work using a standardized workflow (Table 1) [25]. These were human diffuse type gastric cancer, pancreatic adenocarcinoma, high-grade serous ovarian cancer, intrahepatic cholangiocarcinoma, and non-BRCA1/BRCA2 familial breast cancer.

**Table 1:** Twenty exome datasets for five cancer types that were analysed in our previous work for obtaining variant information that led to formation of derivative datasets

Type of cancer	Selected sample files and NCBI SRA IDs
Human diffuse type gastric cancer	SRR941051, SRR941052, SRR941053, SRR941054
Intrahepatic cholangiocarcinoma	SRR894452, SRR900123, SRR900099
High-grade serous ovarian cancer	ERR035487, ERR035488, ERR035489
Pancreatic adenocarcinoma	ERR232253, ERR232254, ERR232255
Non BRCA1/BRCA2 familial breast cancer	ERR166303, ERR166304, ERR166307, ERR166310, ERR166312, ERR166335, ERR166336

The five cancer types were chosen for our initial analysis in our previous studies because they were the major ones affecting the Indian population, for which we aimed to build a model. Although other cancer types such as hepatocellular carcinoma [29, 30], and bone cancer [31, 32] are also significant, the present study focused on model building for the five types as continuation of our previous work. An extension of this work however, will include more cancer types to stabilise the model further. Additionally, variant identification was also performed in our previous work specifically for these five cancer types which were thought to affect the Indian population more.

Moreover, previous studies have shown that no other similar models were available that were built on these five different cancer types, making our method unique. Please refer Padmavathi et al. [25], for more information on the pipeline used and justifications provided for arriving at different variants. These datasets employed for the study are publicly available and can be downloaded from NCBI SRA (https://www.ncbi.nlm.nih.gov/sra) with their accession numbers.

Hyperlinks for the sample files that were employed in our previous work is provided.

#### Data clean-up and obtaining a derived dataset

The given exome dataset consisted of 4181 sample variants, with 88 features. On initial analysis, most of the features were filled with NaN (missing value marker). The initial analysis was done using “Pandas” library module available in python modules. These features were dropped, as they couldn’t be used. The features left were 55 in number. These features were still filled with a few of NaN values. Categorical features with NaN values were dropped as well, since these features were not distinct and filling them with the use of Natural Language Processing (NLP) could not have a significant improvement on the precision prediction of the five types of cancer [33]. These included high-grade serous ovarian cancer, pancreatic adenocarcinoma, human diffuse-type gastric cancer, intrahepatic cholangiocarcinoma and non BRCA1/BRCA2 familial breast cancer. Considering only the numerical features for the prediction model, 25 numerical features were obtained. The few NaN values present in the dataset were filled with probabilistic distribution using probabilistic matrix factorization [34]. These 25 features after handling the missing data over the 4181 sample variants constituted the derived dataset.

#### Exploratory data analysis

Principle Component Analysis (PCA) models were trained over the derived dataset. The number of dimensions in which the dataset was analyzed were one dimension, and two-dimensional axes. The results of the PCA models, reduced the high variance in the dataset due to distributing the weight of the features along two dimensions. Through this distribution the high dimensionality of the dataset was reduced, as the features that would have caused overfitting were removed [35https://colab.research.google.com/drive/1AypJYvigGnpCrhsmLkO6c3b-jSZTqqKN]. The 14 features that had the maximum weight were selected for training the subsequent classification models and were also trained in the ensemble models trained later. The 14 selected features are, ‘shiftscore’ (score for sorting the variants from tolerant to intolerant), ‘TLOD’ (log odds that the variant is present in the tumor sample relative to the expected noise), ‘Sample.AF’ (allelic frequency of the sample), ‘MBQ’ (median base quality of each allele), ‘MFRL’ (median fragment length of each allele), ‘MMQ’ (median mapping quality of each allele), ‘Sample.AD’ (allelic depth of the sample), ‘Sample.F1R2’ (forward and reverse read counts for each allele), ‘Sample.F2R1’ (forward and reverse read counts for each allele), ‘DP’ (read depth), ‘GERMQ’ (phred-scaled posterior probability that the alternate alleles are not germline variants), ‘MPOS’ (median distance from the end of the read for each alternate allele), ‘POPAF’ (population allele frequency of the alternate alleles), and ‘Sample.DP’ (approximate read depth of the sample), (https://support.sentieon.com/appnotes/out_fields/) [36]. These parameters provided information on the variants identified from our previous analysis of cancer exomes, with alleles being the alternative forms of the genes that result from mutations and are present on the chromosomes [37]. Since these parameters were found to be most important that could point towards specific cancer types, these were selected for building our model.

This allowed the authors to reduce the bias-variance trade off that would have been caused due to the use of irrelevant features according to the two-dimensional PCA model [38].

#### Oversampling using SMOTE

**Fig. 2:**
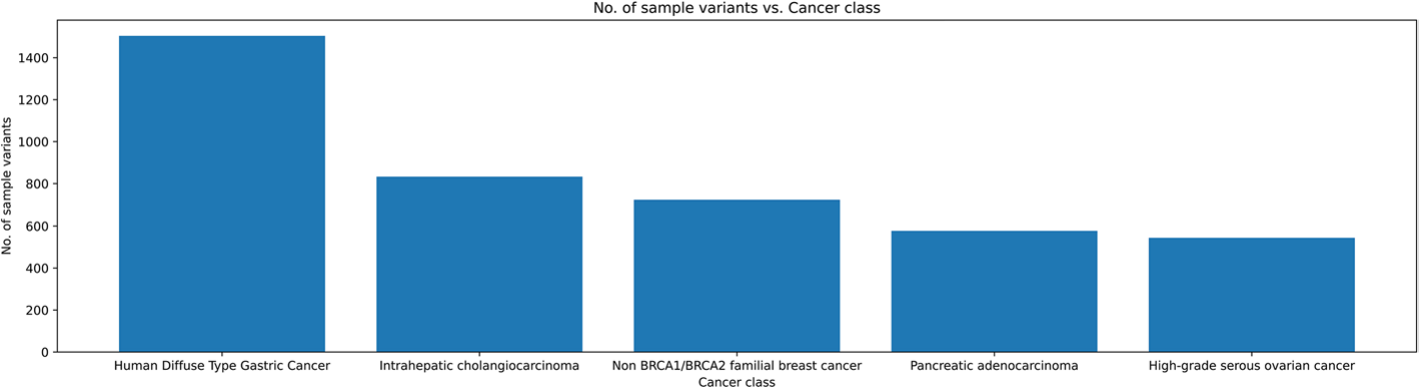
Bar graph representing distribution of exome dataset for types of cancer. From the plot, the cancer class for human diffuse type gastric cancer is in majority by a huge margin compared to other classes. This caused the dataset to be imbalanced

Synthetic Minority Oversampling Technique, also referred to as SMOTE, is an oversampling technique to reduce imbalanced datasets. In the exome dataset, it was found that the dataset was heavily imbalanced with the majority class of cancer being Human diffuse-type cancer having the highest number of sample variant (Fig. 2). This would cause the classifiers to not be sensitive to the change in the features of the dataset [39]. In this technique the minority class types to match the number of sample variants in the majority class type were increased using the SMOTE algorithm. This ensured that the imbalance in the dataset was significantly reduced.

#### Cross validation

Cross validation is a technique used to assess the variance-bias trade-off, of a machine learning model, to understand if the model is overfitting or underfitting, on completely unseen data [40].

The approach followed for cross-validation in our proposed study was hold out cross validation technique. This technique follows by dividing the dataset into a training set and a test set (the test set can be further divided into test and validation set). The model is then trained on the training set, where adjustments are made to its hyper-parameters to balance the variance-bias trade-off. After training the model, the model is subjected to the test set, where all the results produced by the model are considered as a final statement to the performance metrics [40]. This approach was implemented in the present study to cross-validate and confirm the relevance of our model in real-world test scenarios.

### K-nearest neighbors classification model analysis

The K-Nearest Neighbors (KNN) machine learning algorithm is an important pattern recognition-based classifier that has great importance in analyzing and predicting cancer types in exome datasets [41, 42]. The primary step in implementing the KNN classifier is to identify the correct number of clusters that the dataset can be divided into. To identify the correct number of clusters, the elbow-curve method was employed. In this method the KNN classifier using the default hyperparameters, for various values of K, i.e., the number of clusters was applied. The order in which the value of K increases is sequential. Then the error rate versus K-graph is plotted. Through this graph the value of K for which the decrement in error rate is the most significant is chosen as the optimal cluster value, K [43]. After obtaining the optimal cluster value, it was used to train the KNN classifier.

$$D(di,dj) = \sqrt {\frac{1}{N}(\sum (wik - wjk)^{2} )}$$

The above formula, describes the Euclidean distance method, where N is the dimension of the feature vectors, w_k_ is the dimension of the k-th feature vector, and the pair d_i_ and d_j_, denote the feature vector of a specific text in the training set and the feature vector of another text under consideration in the training set [44].

The default hyperparameters relied on using the Euclidean distance to differentiate the data points into different clusters. This did not result in a better classification. To identify the correct hyperparameters, “Grid Search” module was used [45]. From the grid search module, the best hyperparameters were obtained on training the KNN classifier on different hyperparameters using a verbose of 2. The hyperparameters involved using Manhattan distance, reducing the number of leaf nodes, and using “Ball Tree” algorithm over “Brute Force” algorithm. The classification model was then obtained using these hyperparameters.

For two points (x_1_,y_1_), and (x_2_,y_2_), the Manhattan distance can be defined as:

$$\left| {x_{1} - x_{2} } \right| - \left| {y_{1} - y_{2} } \right|$$

where the absolute distance of two points in consideration are calculated. This model is then repeated throughout the different points under consideration for the feature vector present in the dataset, and the classification was carried out [46]. The grid search values are provided in https://colab.research.google.com/drive/1oOBwnfbmy9yLngPSpsJyTCEEOGM_CkmE?usp=sharing#scrollTo=40STvZ9rx8s1 for understanding the range values, which were kept to be a positive integer increment (from 0 to infinity) with verbose of 2.

### Support vector machine classification model analysis

Another popular classification model used for data that can be distinguished better with the use hyperplanes and kernel substitution [47]. In this model the Support Vector Machine (SVM) classifier was used with default hyperparameters on the oversampled dataset. The hyperplanes differentiation can be very well implemented for our dataset, due to the high dimensionality [48].

$$H: w^{T} (x) + b = 0$$

where *H* represents the hyperplane equation, *b* is the bias term of the hyperplane equation, and *w* is the dimension of the feature vector [49].

$$d_{H} (\Phi (x_{0} )) = \frac{{\left| {w^{T} \Phi (x_{0} ) + b} \right|}}{{\left\| w \right\|_{2} }}$$

where the distance function *d* with reference to a point vector, is given in terms of the symbols defined before [49].

Furthermore, “Grid Search” on SVM classifier using “GridSearchCV” to identify the best hyperparameters on a verbose of 2 was performed, but the results of the “Grid Search” module based on the value ranges as follows,

$${\text{`C'}} :[0.{1},\;0.{5},\;{1},\;{5},\;{1}0,\;{15},\;{1}00,\;{15}0,\;{5}00,\;{1}000]$$

$${\text{`gamma'}} :[{1},\;0.{1},\;0.0{1},\;0.00{1},\;0.000{1},\;0.0000{1}]$$

$${\text{`kernel'}} :[{\text{`rbf'}} ,\; {\text{`poly'}} ,\; {\text{`sigmoid'}} ]$$

where ‘*C*’ is the regularization, which acts as a penalty parameter, ‘*gamma*’ defines the suitable line of separation, and ‘*kernel*’(s) are the dimensional modifiers. Within the kernels, ‘*rbf*’ stands for a Gaussian kernel based on standard normal distribution, and the rest ‘*poly*’ and ‘*sigmoid*’ retain their usual meanings.

It was found that the default hyperparameters were best suited for the classification of dataset used in the present study.

### Implementing neural networks

Artificial Neural Networks is a complex system that is designed to function and learn like the human brain [50, 51]. It performs multiple iterations and learns to predict output based on them; it performs backpropagation to update its weight to increase the accuracy of the model. Neural network is able to perform complex analysis and work out the non-linearity between the inputs and the predicted outputs [51–55]. We proposed a 4-layer MLP model with 1D batch normalization and ReLU as the activation function between them; Dropout layers were also added to better train the model. For the classification Neural network criterion selected was Cross entropy loss and Adam [56] was the choice of optimizer.

$$Y_{k} (x) = f\left\{ {\sum\limits_{i = 1}^{n} {\left( {w_{ki} x_{i} + b_{k} } \right)} } \right\}$$

The above equation is used to represent the simplest form of a MLP, i.e., a perceptron, where $Y_{k}$ is the output of the *k*th perceptron and $w_{ki}$ is the *i*th element of the pre-trained weight matrix of *k*th perceptron in any layer. $x_{i}$ is the *i*th input and $b_{k}$ is the bias of the perceptron. As you go down the layer, each output will depend on the output of the previous layers [57].

### Ensemble machine learning approach

Ensemble learning approach involves dividing the dataset into different potential portions, these portions are then given as input to various classifiers, or the same classifiers with different hyperparameters. The ensemble classifiers are stronger classifiers compared to the use of single classifiers, due to the use of weights to judge how well a particular classifier works on a certain or entire portion of the dataset [58]. After development in the field of ensemble learning, the two most widely used algorithms are Bagging (abbreviation for Bootstrap Aggregating) and boosting. These two algorithms have error-correction capabilities, due to which they are predominantly used in training stronger classifiers. In the present study, bagging technique, using the KNN classifier, SVM classifier, and the MLP classifier as the base classifiers was utilized. The weighted average of these three models are calculated and the weights are judged based on majority voting. The ensemble estimator is then employed which calculates the weighted average using a holdout validation set [59–61].

The study was also carried upon “Extended Gradient Boosting techniques (XGBoost) [62]”, but the comparative results with respect to using bagging techniques instead of gradient boosting techniques was mathematically not suitable since, the model would undergo correction to bias and underfitting, instead of focusing on building parallel decision trees which would correct the variance and overfitting by minimizing the same [63]. Therefore, the ensemble estimator was preceded by bagging techniques, instead of utilizing Gradient Boosting Decision Tree (GBDT).

### CTGAN and TVAE implementation on tabular data

Generative Adversarial Networks, or GANs for short, are an approach to generative modeling using deep learning methods, such as convolutional neural networks. Generative modeling is an unsupervised learning task in machine learning that involves automatically discovering and learning the regularities or patterns in input data in such a way that the model can be used to generate or output new examples that plausibly could have been drawn from the original dataset. GAN contain two sub-models: the generator model that we train to generate new examples from noise input and the discriminator model that tried to classify the examples as either real or fake. These two models are trained together in a zero-sum game. CTGAN is a collection of Deep Learning based Synthetic Data Generators for single table data, which are able to learn from real data and generate synthetic clones with high fidelity [64].

Another type of deep generative model is the Variational Autoencoders (VAEs), as the name suggests autoencoder whose encodings distribution is regularized during the training in order to ensure that its latent space has good properties allowing us to generate some new data. The TVAE is model is based on the VAE-based Deep Learning data synthesizer on tabular data, similar to the GAN the VAE contains encoder and decoders instead of generator and discriminator [64].

Under the scheme of the samples that were currently used in the dataset, it was realized that the sample size was insufficient to improve upon the already proposed models as in “K-Nearest Neighbors Classification Model Analysis”, “Support Vector Machine Classification Model Analysis”, “Implementing Neural Networks” and “Ensemble Machine Learning Approach” sections. A novel technique that augments the dataset with increased quantity of samples, simultaneously reducing the imbalance and noise in the dataset, was sought after. The main objective while augmenting the dataset, should be to match clinical trials in terms of correlation. Exploring this novel method will also help reduce the overhead costs and the expenditure on clinical trials due to the expensive instruments used. This would also help in obtaining more conclusive classification in terms of rare types of cancer, for which the sample data could be very small. Although, using SMOTE as mentioned in “Oversampling using SMOTE” section, could be used to increase the sample size, but these augmented datasets are not studied under correlation to the clinical trials. This implies that we do not have a parameter to judge the authenticity of the generated dataset. The overall estimation period of clinical trials often hinders the time taken to proceed with the prediction model. To tackle all these issues, a novel method to augment and increase the sample size of exome dataset using Generative Adversarial Network (GAN) and Triplet based Variational Auto Encoder (TVAE) was proposed [65].

Conditional generator $G(z, cond)$ can be formally described as

$$\left\{ {\begin{array}{*{20}l} {h_{0} = z \oplus cond} \hfill \\ {h_{1} = h_{0} \oplus {\text{ReLU(BN(FC}}_{{{\text{|cond| + |z|}} \to {256}}} { (}h_{{0}} {)))}} \hfill \\ {h_{2} = h_{1} \oplus {\text{ReLU}}({\text{BN}}({\text{FC}}_{{|{\text{cond}}| + |{\text{z}}| + 256 \to 256}} (h_{1} )))} \hfill \\ {{\hat{\upalpha }}_{i} = \tanh ({\text{FC}}_{|cond| + |z| + 512 \to 1} (h_{2} )\quad \quad \quad \quad \quad \;1 \le {\text{i}} \le N_{c} } \hfill \\ {{\hat{\upbeta }}_{i} = gumbel_{0.2} \, (FC_{{|cond| + |z| + 512 \to m_{i} }} { (}h_{{2}} {))}\quad \quad 1 \le {\text{i}} \le N_{c} } \hfill \\ {{\hat{\text{d}}}_{i} = gumbel_{0.2} \, (FC_{{|cond| + |z| + 512 \to D_{i} }} { (}h_{{2}} {))}\quad \quad 1 \le {\text{i}} \le N_{d} } \hfill \\ \end{array} } \right.$$

Discriminator can be formally described as

$$\left\{ {\begin{array}{*{20}c} {\begin{array}{*{20}l} {h_{0} = r_{1} \oplus \cdots \oplus r_{10} \oplus cond_{1} \oplus \cdots \oplus cond_{10} } \hfill \\ {{\text{h}}_{1} = drop(leaky_{0.2} (FC_{{10\left| r \right| + 10\left| {cond} \right| \to 256}} (h_{0} )))} \hfill \\ {{\text{h}}_{2} = drop(leaky_{0.2} (FC_{256 \to 256} ({\text{h}}_{1} )))} \hfill \\ {C( \cdot ) = FC_{256 \to 1} ({\text{h}}_{2} )} \hfill \\ \end{array} } \\ \end{array} } \right.$$

$$- r_{1} \oplus r_{2} \oplus \ldots :notation\;is\;used\;to\;define\;the\;concatenate\;vectors$$

$$- gumbel_{r} (x):apply\;Gumbel\;softmac\;with\;r\;on\;a\;vector\;on\;a\;vector\;x$$

$$- leaky_{r} (x):apply\;a\;leaky\;ReLU\;activation\;on\;x\;with\;leaky\;ratio\;r$$

$$- FC_{u \to v} (x):apply\;a\;linear\;transormation\;on\;u - dum\;input\;to\;get\;a\;v - dim\;output$$

The above equations represent the network structure of CTGAN model; $cond$ vector represents the conditional vector for all of the discrete columns from the dataset which end up as one-hot vectors. $h_{0} , h_{1} \;{\text{and}}\;h_{2}$ represents the output of each layer, while z denotes the input value for the generator. $r_{j}$ is the representation of outputs of each row which is the concatenation of discrete and continuous columns [64, 65].

To overcome the data imbalance, TGAN (Tabular GAN) was implemented on the dataset. But it failed to work on a multi-class classifier as the discrete columns could not be generated. To tackle this issue, Condition Tabular Generative Adversarial Network (CTGAN) model was adopted as the base generation model, which included a generator and a discriminator. The generator and the discriminator were constructed with fully connected layers respectively. The method that was followed was proposed by Xu et al. [64], TVAE stands out to the variational autoencoder (VAE), by reconstructing the features and labels based on the loss of VAE during generating data [64, 65]. This method, however, stands similar to the CTGAN approach in terms of epochs used to generate the tabular data with discrete columns.

### Performance evaluation metrics

Given the true positives (TP), false positives (FP), true negative (TN) and false negative (FN) counts, the following performance evaluation metrics were calculated:

$$Accuracy\user2{ } = \user2{ }\frac{TP + TN}{{TP + FP + TN + FN}}$$

$$Precision\user2{ } = \user2{ }\frac{TP}{{TP + FP}}$$

$$Recall \user2{ } = \user2{ }\frac{TP}{{TP + FN}}$$

$$F_{\upbeta } = \left( {1 +\upbeta ^{2} } \right)\frac{Precision*Recall}{{\left( {\upbeta ^{2} *Precision} \right) + Recall}}$$

The parameters, precision and recall, are based on the relevance of the results retrieved, and help us judge the fraction of relevant instances according to the given mathematical formulae. Accuracy gives us the overall true instances for every reported instance. Although Accuracy guides us through the overall true instances it doesn’t amount to the relevant instances that are important in the present study of prediction of cancer classes [66]. Substituting the values for $\upbeta$ with natural numbers (1, 2, 3, … so on) gives us the corresponding $F_{\upbeta }$ scores, which helps us understand the imbalance in results of large number of actual negatives [66].

Confusion matrix:

A confusion matrix is a table that is used to define the performance of a classification algorithm. It represents counts from the actual and predicted values. All the primary diagonal elements represent the true positives (TP) classifications and the other elements represents the false positives and True negatives. Accuracy can be misleading if used with imbalance datasets, and therefore metrics based on confusion matrix can be more useful and stable comparatively [67]. This concept was implemented in our study where confusion matrices were drawn to represent the probabilities of true and false positives for 5 cancer types.

## Results

### Comparison of KNN and SVM classifiers

**Fig. 3:**
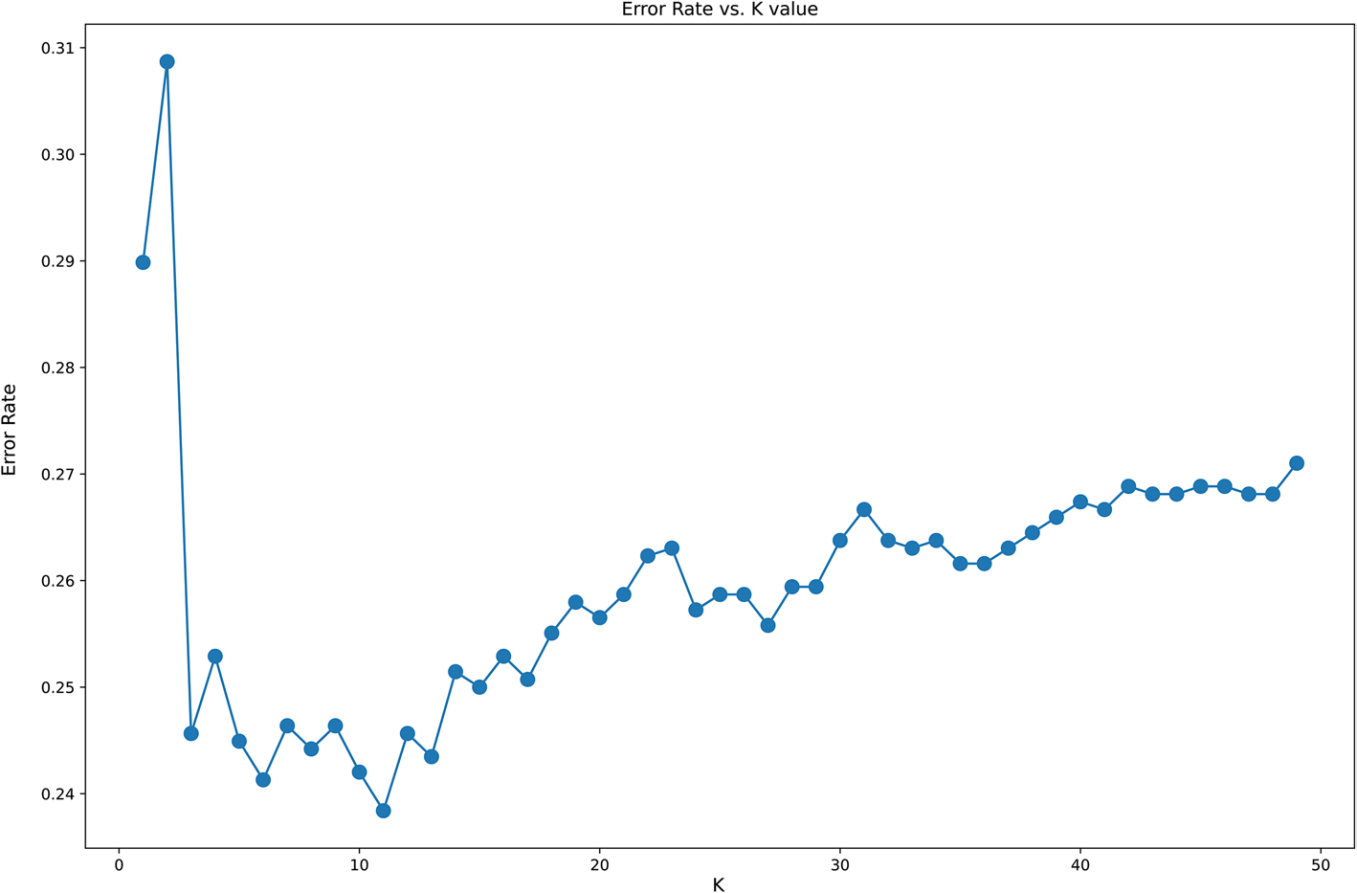
Distortion (error rate) versus number of clusters (K-value) generated by elbow curve method to determine the best K-value for KNN. From the graph, we can see that the error rate drastically drops around 14, thereafter the decrease in error rate is low. Therefore, the ideal cluster value should be between 11 and 13

After performing the elbow curve method to identify optimal number of clusters (Fig. 3), the models classification report obtained using KNN classifier had a weighted average of 0.74. But, the precision, recall and f1-score for cancer types of high-grade serous ovarian cancer, and pancreatic adenocarcinoma, were very low, with precision resulting in 0.59 and 0.58 respectively. But the precision value for other three types of cancer were above 0.75, that lead to the understanding that the cancer types with lower precision and recall were affected by the high dimensionality of the dataset. The hyperparameters used in KNN classifier as mentioned in "K-nearest neighbors classification model analysis" section, gives better true positives as compared with the default hyperparameters. The weighted accuracy using the KNN classifier with the default hyperparameters was 0.69, whereas with the selected hyperparameters, the weighted accuracy increased to 0.77 (Table 2a) The results and code can be found here https://colab.research.google.com/drive/1FNv8jKhT9o2zJ7s1UtwsNO3S_K306ZAz.

**Table 2:** Performance metrics for KNN, SVM, MLP and ensemble classifiers for five cancer types

Classifier	Cancer class	Precision	Recall	F1-score
(a) Performance metrics for KNN				
KNN	High-grade serous ovarian cancer	0.56	0.61	0.59
	Human diffuse type gastric cancer	0.88	0.71	0.79
	Intrahepatic cholangiocarcinoma	0.79	0.85	0.82
	Non BRCA1/BRCA2 familial breast cancer	0.82	0.96	0.89
	Pancreatic adenocarcinoma	0.60	0.62	0.61
Weighted accuracy		0.77		
(b) Performance metrics for SVM				
SVM	High-grade serous ovarian cancer	0.66	0.58	0.62
	Human diffuse type gastric cancer	0.83	0.66	0.73
	Intrahepatic cholangiocarcinoma	0.85	0.86	0.86
	Non BRCA1/BRCA2 familial breast cancer	0.84	0.99	0.91
	Pancreatic adenocarcinoma	0.62	0.71	0.66
Weighted accuracy		0.76		
(c) Performance metrics for neural networks				
Neural networks	High-grade serous ovarian cancer	0.75	0.74	0.74
	Human diffuse type gastric cancer	0.83	0.78	0.80
	Intrahepatic cholangiocarcinoma	0.85	0.89	0.87
	Non BRCA1/BRCA2 familial breast cancer	0.89	0.92	0.91
	Pancreatic adenocarcinoma	0.78	0.78	0.78
Weighted accuracy		0.82		
(d) Performance metrics for ensemble model				
Ensemble model	High-grade serous ovarian cancer	0.76	0.78	0.77
	Human diffuse type gastric cancer	0.82	0.77	0.79
	Intrahepatic cholangiocarcinoma	0.84	0.91	0.87
	Non BRCA1/BRCA2 familial breast cancer	0.89	0.93	0.91
	Pancreatic adenocarcinoma	0.83	0.77	0.80
Weighted accuracy		0.82		

Additionally, increasing the cluster value (K) didn’t have any significant effect on the classification report, leading to a very small decrement in the value of weighted average to 0.68. To tackle the issue of high dimensionality and to improve the classification report for the cancer types, high-grade serous ovarian cancer, and pancreatic adenocarcinoma, the classifier was switched to SVM. As mentioned in "Support vector machine classification model analysis" section, the SVM classifier using the default hyperparameters performed much better in overall classification report for all the five cancer types. The weighted average remained around 0.76 (Table 2b), with the precision for cancer types, high-grade serous ovarian cancer, and pancreatic adenocarcinoma, improving to 0.66 and 0.62 respectively (Table 2b) (https://colab.research.google.com/drive/1FEJBNzT8wYYwEKpYsaqFuq_TyhzsKaA7). The value of true positives almost doubled, but the downfall was the value of false negatives that had slightly increased.

### Neural networks performance

**Fig. 4:**
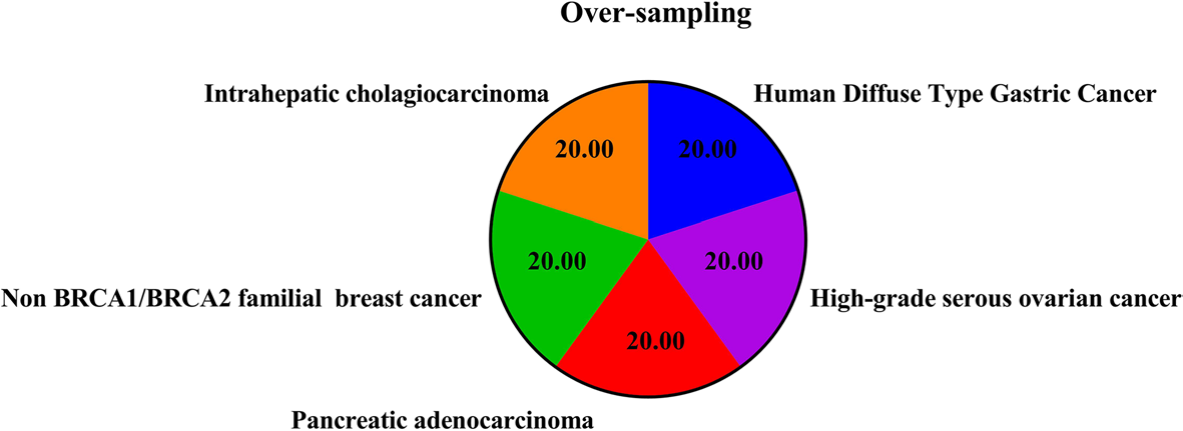
Balanced dataset using oversampling via SMOTE. This plot shows that the all the cancer class is equally balanced after performing SMOTE oversampling and should make the model trained to be more stable

**Fig. 5:**
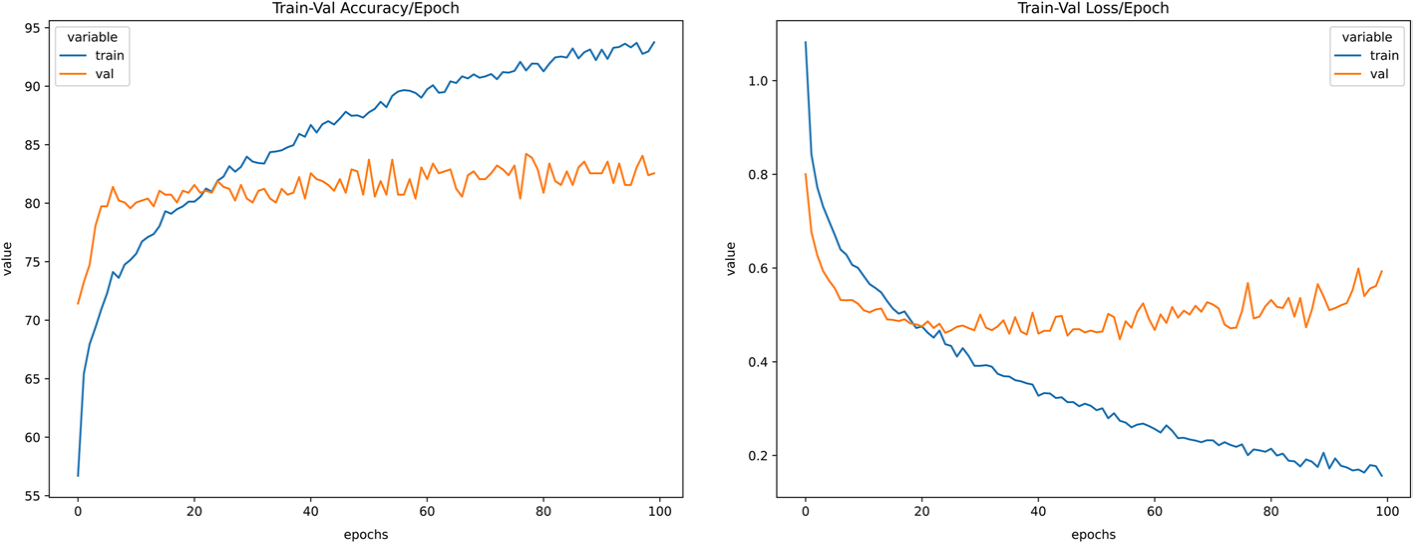
Train-validation accuracy versus epochs and train-validation loss versus epochs for neural network with SMOTE oversampling. From the graph, it can be seen that the validation accuracy stalls around 40 epochs and has only slight variation after that hence training for 40 epochs should be sufficient to provide same performance as training for 100 epochs. From the validation loss graph, it is noted that after around 50 epochs the model starts to overfit for the training data and hence stopping it after that should prevent it from overfitting

**Fig. 6:**
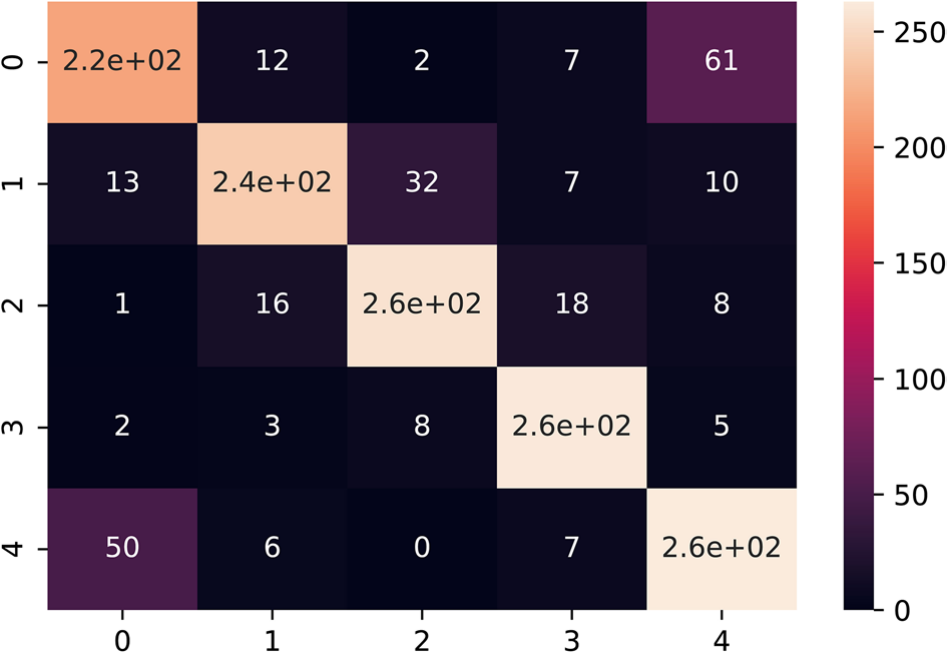
Confusion matrix heatmap of neural network with SMOTE oversampling. The primary diagonal elements from this graph shows the true correct positives and the rest are the false classification. Higher number of primary diagonal from the matrix shows that the classifier has achieved a good accuracy. 0–4 represents the five cancer classes. 0: High-grade serous ovarian cancer, 1: Human diffuse-type gastric cancer, 2: Intrahepatic cholangiocarcinoma, 3: Non BRCA1/BRCA2 familial breast cancer, 4: Pancreatic adenocarcinoma. The light to dark color coding indicates the probabilities of true and false positives

**Fig. 7:**
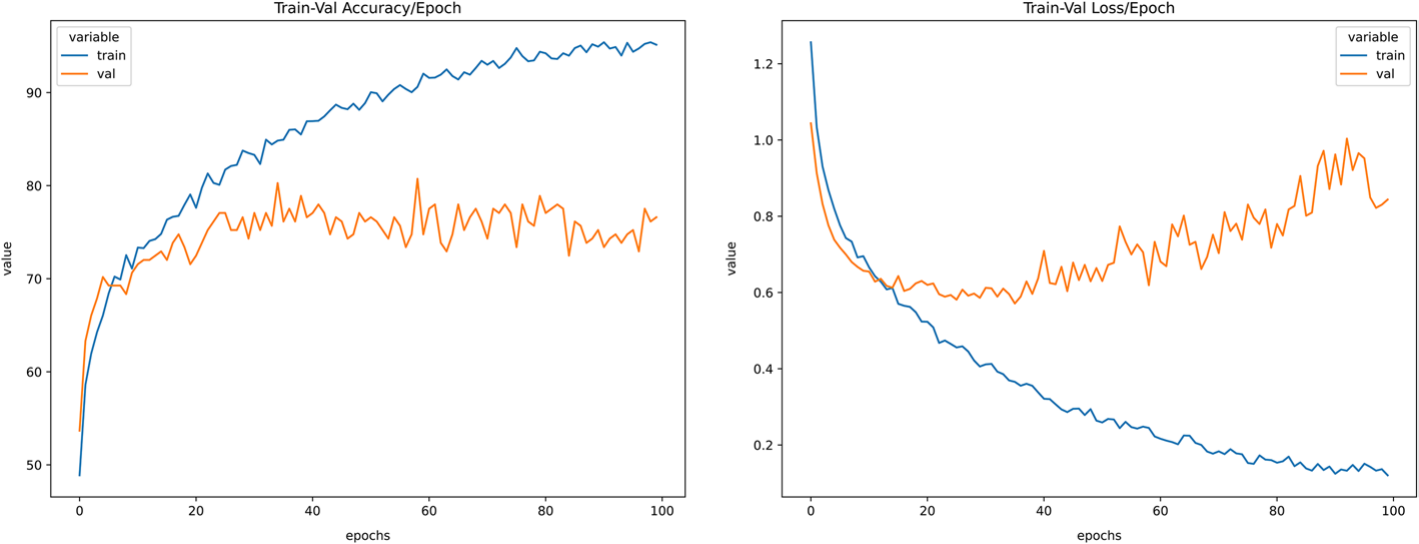
Train-validation accuracy versus epochs and train-validation loss versus epochs for neural network with under sampling. From the graph, it can be seen that the validation accuracy stalls around 40 epochs and has only slight variation after that hence training for 40 epochs should be sufficient to provide same performance as training for 100 epochs. From the validation loss graph, it is noted that after around 40 epochs the model starts to overfit for the training data and hence stopping it after that should prevent it from overfitting

**Fig. 8:**
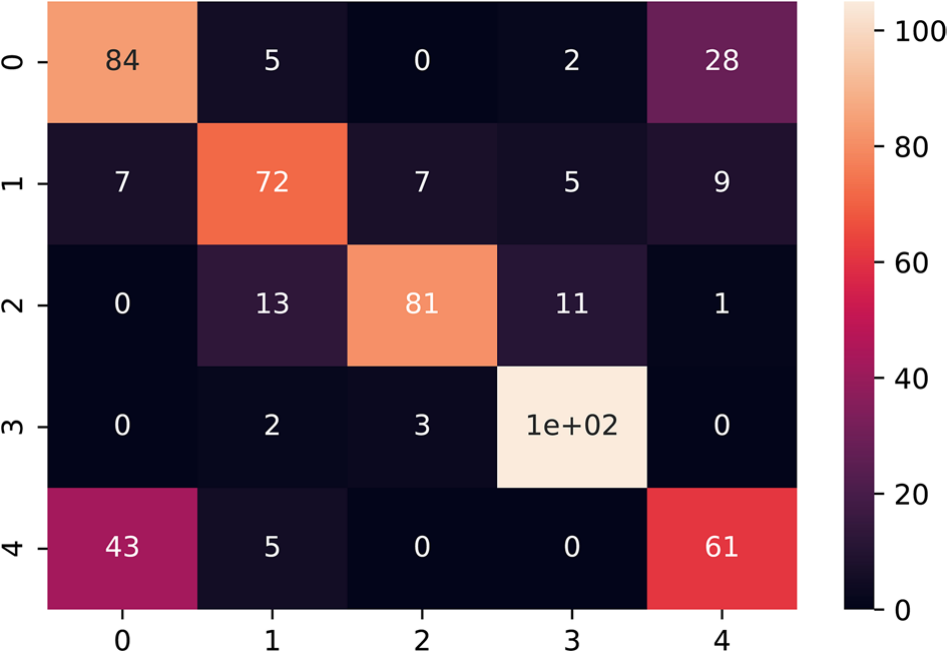
Confusion matrix heatmap of neural network with undersampling. The primary diagonal elements from this graph shows the true correct positives and the rest are the false classification. Higher number of primary diagonals from the matrix shows that the classifier has achieved a good accuracy but performance was worse compared to SMOTE oversampling. 0–4 represents the five cancer classes. 0: High-grade serous ovarian cancer; 1: Human diffuse-type gastric cancer; 2: Intrahepatic cholangiocarcinoma; 3: Non BRCA1/BRCA2 familial breast cancer; 4: Pancreatic adenocarcinoma. The light to dark color coding indicates the probabilities of true and false positives

The trained model was experimented with under sampled and SMOTE oversampled dataset (Figs. 4, 5, 6, 7, 8). The model was trained for 100 epochs with a batch size of 20 in custom balanced batches. On the under sampled, the model had a weighted average of 0.73. The precision obtained for Intrahepatic cholangiocarcinoma was 0.88 and for Non BRCA1/BRCA2 familial breast cancer was 0.86. The precision for three of the cancers—High-grade serous ovarian cancer, Human Diffuse Type Gastric Cancer and Pancreatic adenocarcinoma was 0.62, 0.66 and 0.66 respectively which was significantly lower. The model also showed wrong classification between High-grade serous ovarian cancer and Pancreatic adenocarcinoma which could be traced to less data due to under sampling. This model had validation accuracy of 74.31% after 100 epochs and average accuracy of 73% on the test set.

On the SMOTE oversampled dataset, the individual precision of the above 3 cancers increased significantly; High-grade serous ovarian cancer increased to 0.75, Human Diffuse Type Gastric Cancer increased to 0.83 and Pancreatic adenocarcinoma increased to 0.78. The precision obtained for Intrahepatic cholangiocarcinoma was 0.85 and for Non BRCA1/BRCA2 familial breast cancer was 0.89. This model showed 82.56% validation accuracy after 100 epochs and average accuracy of 82% (Table 2c) on the test set. This model showed to be more stable that the one trained on under sampled dataset and increased the precision and recall for all the types of cancer. Results and codes for the same can be found here https://colab.research.google.com/drive/1lH2tdApkHfqF_6C-d9Pe3o2ZR6oCjp-5, https://colab.research.google.com/drive/1KSDKoxJmbNwW_hBElV2DP-CIlLDA-eP0.

### Weighted ensemble learning classifier

As discussed in “Ensemble Machine Learning Approach” section, the base classifiers identified to be ideal had to be weighted according to their performance on the classification of the cancer types. To perform this function, the “tensordot” API available in the “NumPy” module was used (https://numpy.org). The tensordot API helps in calculating the tensor product of the weighted accuracy obtained from the base classifiers. The weighted accuracy of the KNN classifier, SVM classifier, and MLP classifier were 0.754, 0.774, and 0.842 respectively. The ensemble classifier had a weighted accuracy of 82.91% (Table 2d). The dataset was divided into 70:15:15 ratio. The 70:15 was used to split into training and test sets. The remaining 15% was used for the holdout validation set. The performance metric was calculated by fitting the test set to the base classifiers, and then measuring the true positives using majority voting. Using only KNN and SVM classifiers as base classifiers the weighted accuracy of the ensemble estimator still performed better with soft voting, resulting in 78.288%. In this case, the KNN classifier and SVM classifier models had weighted accuracy of 0.736 and 0.701 respectively (https://colab.research.google.com/drive/1mFcOy--VT1hQem8JhClh5TfSK5KnLKJL). The confusion matrix from the resulting ensemble classifier (Table 3), had much better evaluation metrics, with the precision value for high grade serous ovarian cancer, and pancreatic adenocarcinoma reaching 0.76 and 0.83, compared to the results in "Comparison of KNN and SVM classifiers" section. The entire results have been depicted in Table 4, where the performance parameter used for the results is precision. The justification for choosing such a parameter is to allow the weightage of false positives (FP), to have a greater ratio in determining the results as from the statistical relation in "Performance evaluation metrics" section, we observe precision to give us a significant ratio for the same. The weightage of false positives, helps us in the case of prediction of cancer classes based on exome dataset. Precision, has been selected as the required performance metric, as the requirement of having a better ratio in false positives (FP), has a greater significance in cancer prediction for a decision support system. The table therefore, summarizes our proposed models and their respective precision values. The results are presented in SOTA method.

**Table 3:** Confusion matrix from resulting ensemble classifier

Confusion matrix*	0	1	2	3	4
0	302	98	0	36	105
1	58	1425	92	134	93
2	0	117	630	110	14
3	3	15	22	928	3
4	79	137	21	34	544

*0–4 indicate cancer classes high-grade serous ovarian cancer, human diffuse type gastric cancer, intrahepatic cholangiocarcinoma, non BRCA1/BRCA2 familial breast cancer, pancreatic adenocarcinoma respectively

**Table 4:** Results based on precision for the proposed classifiers under study

Cancer classes	Classification algorithms (precision)			
	KNN	SVM	Neural network	Ensemble
High-grade serous ovarian cancer	0.56	0.66	0.75	0.76
Human diffuse type gastric cancer	0.88	0.83	0.83	0.82
Intrahepatic cholangiocarcinoma	0.79	0.85	0.85	0.84
Non BRCA1/BRCA2 familial breast cancer	0.82	0.84	0.89	0.89
Pancreatic adenocarcinoma	0.60	0.62	0.78	0.83

### CTGAN and TVAE generated dataset

The proposed model for CTGAN was trained for 300 epochs with a batch size of 10 after which the generator loss was 0.2503 and the Discriminator loss was − 1.4397. The synthetic dataset on evaluation with real dataset with CSTest and KSTest the evaluation metric value was 0.92 and the overall comparison value was 0.66. The proposed TVAE model was also trained for 300 epochs with a batch size of 10 (https://colab.research.google.com/drive/1mFcOy--VT1hQem8JhClh5TfSK5KnLKJL). The synthetic dataset on evaluation with real dataset with CSTest and KSTest, the evaluation metric was 0.93 and the overall comparison value was 0.63.

## Discussion

### Ensemble learning technique

In the ensemble learning algorithm used in the present study, the ensemble estimator to perform soft voting on all the respective base classifiers that were used was proposed. The difference between soft voting and its alternate, hard voting, is that the latter works on the principle of majority label that was classified by all the base classifiers. Whereas soft voting relies on the base classifiers generating a probability value for the target class. From "Weighted ensemble learning classifier" section, the use of soft voting was employed so as to allow each classifier to be judged for every class according to its performance, and then adding the tensor sum. The target label with the greatest total of the weighted probabilities gets the vote [68, 69]. In Assiri et al. [70] the ensemble learning model proposed on the hard voting mechanism had shown better accuracy reaching 99.42%. The proposed classifiers were simple logistic regression learning, support vector machine learning with stochastic gradient descent optimization and multilayer perceptron network. This works on classification of single type of cancer class, i.e., the cancer class under study in Assiri et al. [70], was breast tumor classification on dataset taken from the Wisconsin Breast Cancer Dataset (WBCD). In the model proposed in the present study, the classification of five types of cancers simultaneously was enhanced with ‘Non BRCA1/BRCA2 familial breast cancer’, also a class under study, yielding a recall value of 0.92 and precision of 0.89. From Table 5, using SVM learning with stochastic gradient descent (SGD) optimization the recall and precision were 0.979 and 0.978 respectively. This leads to the inference that SVM with SGD would be a better parameter, but this would be inaccurate due to the fact that breast tumour classification in Assiri et al. [70], have parameters such as the radius of curvature, which can be correctly classified using a gradient descent in a hyperplane; but would be incapable to do so for features that belong only to the exome dataset, as using the same models led to a decrease in precision for ‘Non BRCA1/BRCA2 familial breast cancer’ in our proposed study. Similarly, Table 5 depicts the other 3 proposed models and their respective performance evaluation metrics. The ensemble model based on majority voting described in Assiri et al. [70], plateaus around 0.994. Comparing the performance evaluation metrics in Table 6, from our proposed study we see from the results in "Comparison of KNN and SVM classifiers" section, the recall value for the cancer class ‘Non BRCA1/BRCA2 familial breast cancer’, to be at a high 0.99 in case of SVM using the hyperparameters discussed in "Support vector machine classification model analysis" section, i.e., the default hyperparameters. And has a recall value of 0.96, and 0.92 in case of K-Nearest Neighbour and Neural Networks respectively, as depicted in the Table 6. The majority-based ensemble method developed for all the 5 cancer class in our proposed study, resulted in a recall value of 0.93 for the ‘Non BRCA1/BRCA2 familial breast cancer’ as depicted in Table 6. This, clearly leads to the conclusion that for exome dataset, our proposed ensemble model had better relevant results compared to Adel S. Assiri et al. [70].

**Table 5:** Classification analysis by Assiri et al. [70]

Classification algorithms	Accuracy (%)	Precision	Recall	F1 score
Simple logistic regression learning	98.25	0.983	0.982	0.982
SVM learning with SGD optimization	97.88	0.979	0.978	0.971
Multilayer perceptron network	97.66	0.977	0.977	0.977
K-nearest neighbor classification	97.08	0.972	0.971	0.972
Majority based ensemble model	99.42	0.994	0.994	0.994

**Table 6:** Non BRCA1/BRCA2 familial breast cancer

Classification algorithms	Precision	Recall	F1 score
K-nearest neighbor	0.82	0.96	0.89
Support vector machine	0.84	0.99	0.91
Neural networks	0.89	0.92	0.91
Majority based ensemble model	0.89	0.93	0.91

In this model, soft voting was used to counter the fact, that from "Comparison of KNN and SVM classifiers" section, it was clear that the five cancer types were not well distinguished, simultaneously by the KNN or SVM classifier. Using soft voting instead of hard, allowed us to predict the cancer class better by giving each of the individual classifiers a probability value based on their performance with the holdout validation set. From "Weighted ensemble learning classifier" section, the weighted accuracy of the model was found to be 82.91%. Furthermore, on training the ensemble estimator using hard voting, i.e., majority voting, the overall weighted accuracy was observed to be 76.758%.

In Li et al. [71], the reported overall accuracy was 71.46% for the classification of 14 types of cancer class with the use of performance weighted voting ensemble on five classifiers, logistic regression, support vector machine, random forest, XGBoost and neural networks. From Table 7, the overall weighted accuracy for 8-cancer types calculated for the five classifiers mentioned above, was well below 70% [71]. Only the performance weighted voting ensemble model resulted in an overall accuracy of 71.46 [71]. This clearly shows that the ensemble model with performance weighted voting for greater number of classifiers doesn’t yield significant results, as it is necessary to define a distinguishable structure for the exome dataset by including hyperplane distinction. From Table 8, the weighted accuracy in all cases of different classifiers used in our proposed study is greater than 76%, with the ensemble model based on soft-voting resulting in 82% weighted accuracy. Furthermore, the recall values of the models proposed in our study were significantly higher for all the 5 cancer types. Our proposed model however, resulted in much better overall accuracy of 83%, with the evaluation parameters outperforming the model based on performance weighted voting ensemble.

**Table 7:** Performance evaluation metric, Li et al. [71], for 8 cancer types

Classification algorithms	Accuracy
Logistic regression	0.68
SVM	0.63
Random forest	0.54
XGBoost	0.62
Neural network	0.68
Performance-weighted-voting	0.71

**Table 8:** Performance evaluation metric, proposed study for 5 cancer types

Classification algorithms	Weighted accuracy
KNN	0.77
SVM	0.76
Neural networks	0.82
Majority voting ensemble	0.83

Furthermore, from their research soft voting model had the overall accuracy output comparatively lesser than that of the performance model. However, from the present research the soft-voting ensemble model performed much better as compared the performance model, due to the three classifiers that were used (as mentioned in "Weighted ensemble learning classifier" section), being able to distinguish and give better probability values as compared to the five weak classifiers used in Li et al. [71]. The model designed in the present work also resulted in much larger true positives, and hence a better method for the early prediction of 5 classes of cancer as mentioned in "Data clean-up and obtaining a derived dataset" section.

Additionally, Tables 6 and 8, refer to soft voting classifiers in majority voting ensemble, which use predicted probabilities for class labels, and give almost proportional contribution to predictions for all the involved models. Table 7, pertains to the performance weighted voting ensemble model used in Li et al. [71], and involves a non-uniform weight attached to the models based on different judging parameters. Therefore, the model under Li et al. [71], (Table 7) and the soft-voting models in Tables 6 and 8 are different.

### CTGAN and TVAE on synthetic dataset

The synthetic dataset obtained from CTGAN and TVAE, was saved as a comma separated value file (csv). The proposed ensemble learning model was carried out on the synthetic dataset generated by the CTGAN method (Additional file 1) [72]. The weighted accuracy of the model was about 63.54%, with recall values and precision values for the cancer classes being low. This however was not the case with synthetic dataset generated through TVAE (Additional file 2). On training with the proposed ensemble model, the weighted accuracy was observed to be about 76.58%, with very good recall and precision values. But the main objective of the generated dataset was to be able to distinguish between the cancer classes with lower probability values of being classified. This was easily observed in the model that was trained on TVAE synthetic generated dataset, with very good recall values (Table 9). Clearly, using TVAE and CTGAN can be proposed for improving the oversampling, as well as improving the resultant true positives and false positives. This has a great importance in saving resources, and improving the prediction probability, as compared to other oversampling techniques such as SMOTE.

**Table 9:** Ensemble model trained on TVAE generated dataset

Classifier	Cancer class	A	B	C	D	E
	Evaluation metrics					
Ensemble model	Precision	0.68	0.80	0.82	0.75	0.72
	Recall	0.56	0.79	0.72	0.97	0.67
	F1-score	0.61	0.79	0.77	0.84	0.69
	Weighted accuracy	0.765				

A: High-grade serous ovarian cancer; B: Human diffuse-type gastric cancer; C: Intrahepatic cholangiocarcinoma; D: Non BRCA1/BRCA2 familial breast cancer; E: Pancreatic adenocarcinoma

## Conclusion

The present research work has important clinical significance for identifying the origin of five cancer types and provides insight on obtaining better cancer risk probabilities for the five selected types. In this paper, various algorithms were explored on the exome dataset to classify the cancers. In addition, the present work presented an ensemble machine learning method to combine the benefits of the 3 models (KNN, SVM and Neural network) into one model to provide a more balanced cancer classifier to obtain more accurate predictions. When KNN and SVM models were used, the weighted accuracy using the KNN classifier with the default hyperparameters was 0.69, whereas with the selected hyperparameters, the weighted accuracy increased to 0.77. Likewise, the SVM classifier using the default hyperparameters performed much better in overall classification report for all the five cancer types. The weighted average remained around 0.76. With the neural networks model, the model had validation accuracy of 74.31% after 100 epochs and average accuracy of 73% on the test set. However, with SMOTE on the datasets, the model showed 82.56% validation accuracy after 100 epochs and average accuracy of 82% on the test set. This model showed to be more stable that the one trained on under sampled dataset and increased the precision and recall for all the types of cancer. With the ensemble classifier model, the accuracy upped to 82.91%, close to 83% proving that this model improved the overall model precision.

The trained models enabled us to understand the impact of TVAE on the generation of datasets, by reducing the false negatives by a considerable amount. From the realization of bagging techniques in ensemble machine learning and utilizing weighted ensemble learning technique using soft-voting, the cumulative results yielded a better overall model collection consisting of the same explained throughout "Ensemble learning technique" and "CTGAN and TVAE on synthetic dataset" sections. The classifications obtained through Tables 8 and 9, both provide insight into the mathematical understanding of how the exome datasets can be better partitioned and studied in a hyperplane, as well as distributing the values of the dataset through TVAE and CTGAN, allows us to understand the distribution of the generated datasets as well. Hence, proving to be a vital technique to build a correction system for all types of classifications and reduce the bias-variance trade off which was studied throughout "Weighted ensemble learning classifier" and "CTGAN and TVAE generated dataset" sections.

Further enhancement is dependent on the addition of more variation data from other cancer types. Moreover, the model developed in this work also incorporated study on under sampling, over sampling for data balancing and a novel approach of data augmentation using CTGAN and TVAE was added to the model which proved to be effective in rare cancer cases where data is not widely available, hence proving data similar to real world samples.

## Supplementary Information

**Additional file 1**. The proposed ensemble learning model carried out on the synthetic dataset generated by the CTGAN method.

**Additional file 2**. The synthetic dataset generated through TVAE method.

## Data Availability

All data generated or analysed during this study are included in this published article and its supplementary information files. The derivative datasets used in the current study are generated from analysis of datasets downloaded from publicly available NCBI SRA database. The below NCBI SRA datasets were used in our previous work, Padmavathi et al. [25] and Padmavathi et al. [26] to arrive at data that was used in the current study. SRR894452, SRR900123, SRR900099, SRR941051, SRR941052, SRR941053, SRR941054, ERR166303, ERR166304, ERR166307, ERR166310, ERR166312, ERR166335, ERR166336, ERR035487, ERR035488, ERR035489, ERR232253, ERR232254, ERR232255. The proposed ensemble learning model carried out on the synthetic dataset generated by the CTGAN method is available in Additional file 1. The synthetic dataset generated through TVAE is available in Additional file 2.

## Abbreviations

DSS: Decision support system
NLP: Natural language processing
PCA: Principal component analysis
SMOTE: Synthetic minority oversampling technique
KNN: K-nearest neighbour
SVM: Support vector machine
MLP: Multi-layer perceptron
GAN: Generative adversarial networks
TVAE: Triplet based variational auto encoder
CTGAN: Conditional tabular generative adversarial networks
